# Association of Chewing Tobacco and the Risk of Breast Cancer in Indian Women: A Multicentre Case‐Control Study

**DOI:** 10.1155/ijbc/2950851

**Published:** 2026-03-09

**Authors:** Romi Moirangthem, Ankita Manjrekar, Gayathri B. Pullat, Shruti Vishwas Golapkar, Ruchita Sahdev Margale, Sakshi Sagare, Dipakshi Talukdar, Nandini Chakraborty, Ruchi Pathak, Satyajit Pradhan, Manigreeva Krishnatreya, Nandkumar Panse, Rajesh Dikshit, Isabelle Soerjomataram, Sudeep Gupta, Sharayu Mhatre

**Affiliations:** ^1^ Division of Molecular Epidemiology and Population Genomics, Centre for Cancer Epidemiology, Tata Memorial Centre, Navi Mumbai, Maharashtra, India, tmc.gov.in; ^2^ Homi Bhabha National Institute (HBNI), Mumbai, Maharashtra, India, hbni.ac.in; ^3^ Department of Medical and Paediatric Oncology, Dr. B. Borooah Cancer Institute-Tata Memorial Centre, Guwahati, India; ^4^ Clinical Research Secretariat, Dr. B. Borooah Cancer Institute-Tata Memorial Centre, Guwahati, India; ^5^ Department of Epidemiology and Community Screening, Mahamana Pandit Madan Mohan Malviya Cancer Centre, Varanasi, Uttar Pradesh, India; ^6^ Mahamana Pandit Madan Mohan Malviya Cancer Centre, Varanasi, Uttar Pradesh, India; ^7^ Department of Cancer Registry and Epidemiology, Dr. B. Borooah Cancer Institute-Tata Memorial Centre, Guwahati, India; ^8^ Division of Molecular Epidemiology and Population Genomics, Centre for Cancer Epidemiology, Nargis Dutt Memorial Cancer Hospital, Solapur, Maharashtra, India, tmcepi.gov.in; ^9^ Centre for Cancer Epidemiology, Tata Memorial Centre, Navi Mumbai, Maharashtra, India, tmc.gov.in; ^10^ Cancer Surveillance Branch, International Agency for Research on Cancer (IARC), World Health Organization, Lyon, France, who.int; ^11^ Director Tata Memorial Centre, Tata Memorial Centre, Mumbai, Maharashtra, India, tmc.gov.in

**Keywords:** breast carcinoma, smokeless tobacco, spit tobacco

## Abstract

**Background:**

Even though breast cancer (BC) is the most common female cancer worldwide, the role of tobacco, specifically chewing tobacco in the development of BC has not been widely studied. This study is aimed at assessing this association.

**Methods:**

A multicentre hospital‐based case‐control study was utilised. Two thousand five hundred fifty‐three histopathologically confirmed BC cases, and 2239 visitor controls were included. Self‐reported information was collected regarding tobacco consumption and other potential confounders. A logistic regression model was used to calculate odds ratio (OR) and its 95% confidence interval (CI), after adjusting for age, current residential region, education, various reproductive factors, BMI and history of benign breast lump. Attributable fraction (AF) and population attributable fraction (PAF) of BC due to chewing tobacco were also calculated.

**Results:**

An increased risk of BC was observed in women who ever used chewing tobacco (OR:1.19, 95% CI:1.00‐1.41) as compared to those who never consumed tobacco (smoking and chewing), consistent across all subtypes of BC. A dose‐response relation was observed for duration of tobacco chewing (OR_>25 years_: 1.38, 95% CI: 1.04–1.83). Women who initiated chewing tobacco at < 20 years, before their first full‐term pregnancy (FFTP), had more risk. Observed association was consistent even after stratification on menopausal status. The AF of BC due to tobacco chewing in our study was calculated to be approximately 3%, whereas the PAF for India was about 2%.

**Conclusion:**

Our study suggests that chewing tobacco is associated with an increased risk of BC for all subtypes. This is particularly true when the duration of exposure is higher and exposure begins before FFTP. This highlights the need to target tobacco control policies to smokeless tobacco along with smoking, thus reducing the burden of BC to some extent.

## 1. Introduction

The consumption of tobacco is a major public health concern in India and is linked to many chronic diseases including cancer. While smoking tobacco is a global concern, the use of smokeless tobacco (SLT), particularly chewing of tobacco, is a common practice only in a few South and Southeast Asian countries like India, Bangladesh, Pakistan and Sri Lanka [1]. In India, the prevalence of SLT use exceeds that of smoking tobacco (21.4% vs. 10.7%, respectively) [2]. Among Indian women, smoking is relatively uncommon, with a prevalence of just 1.1% [3]; however, the prevalence of chewing tobacco is estimated to be 11.53%, far exceeding the global prevalence of 2.9% [4]. Tobacco use contributes to around 1.35 million deaths annually in India [5], and is one of the leading causes of cancer incidence and related deaths [6]. The International Agency for Research on Cancer (IARC) has listed tobacco as a cause of at least 14 cancer types, including cancers of the oral cavity, lungs, larynx, and urinary bladder among others [7].

Although breast cancer (BC) is the most common cancer site amongst women worldwide [8], the role of tobacco in the development of the disease has not been widely studied since it was not considered a tobacco‐related cancer. However, emerging evidence suggests that chemicals contained in tobacco can trigger the development of BC [9]. Tobacco products, including SLT products, contain carcinogenic substances like N ^′^‐nitrosonornicotine (NNN) and 4‐(methylnitrosamino)‐1‐(3‐pyridyl)‐1‐butanone (NNK), etc., which can induce carcinogenesis in breast tissues by forming DNA adducts that may lead to cancer‐causing mutations [10], and may also elevate local oestrogen production, which in turn increases the risk of BC.

While the relationship between smoking tobacco and BC have been explored [9, 11, 12], limited literature have examined the role of SLT, specifically chewing tobacco in the risk of developing BC. Preliminary studies in this regard suggest a potential positive association [13–15]. Given the high burden of BC in India (age‐standardised rate of 26.6 per 100,000) as well as high prevalence of chewing tobacco amongst women in India, it is critical to assess the role of this potentially preventable risk factor.

This study is aimed at identifying the association between chewing tobacco and the risk of BC in Indian women, using data from a multicentre hospital‐based case‐control study. More precisely, this study assesses the association of chewing tobacco, its duration of use, age at initiation, and time of initiation defined by reproductive events, with the risk of BC. We further investigated whether the risk differs between premenopausal and postmenopausal women, different molecular subtypes of BC as well as across different BMI categories.

## 2. Materials and Methods

### 2.1. Study Design and Study Participants

A hospital‐based case‐control study was conducted across various hospitals of Tata Memorial Centre (TMC), Mumbai, India and Nargis Dutt Memorial Cancer Hospital, Barshi, Maharashtra, India. The study was carried out in two distinct phases, with participant enrolment occurring from 2008–2016 in the first phase and from 2019–2023 in the second phase. The source population for the study was the respective hospitals. All females aged 20–69 years, residents of India for at least a year, coming to the hospitals (both patients and visitors) were eligible to participate in the study. All the eligible participants were approached by trained interviewers; written informed consent was taken from those who agreed to participate in the study.

Cases were histopathologically confirmed patients with primary carcinoma of the breast (International Classification of Diseases for Oncology: C50.0 to C50.9). Their date of diagnosis did not cross 6 months from the date on which they were enrolled (Supplementary figure 1). Controls were selected from the source population from where cases were enrolled. Controls comprised visitors of the patients and were enrolled simultaneously with cases. They were frequency matched to cases for age (± 10 years) and current region of residence (north, east, west, south and central). Individuals who had personal history of cancer diagnosis were not included as controls. It was also ensured that the controls were not related to patients with any specific cancer site. For this, it was made sure that any specific disease management group—units that specialise in treating specific cancer sites in the hospital (e.g., BC cases are treated under the Breast DMG) did not contribute to more than 30% (Table S1). Females who were pregnant or breastfeeding at the time of enrolment were excluded from the study. The non‐response rate among cases was approximately 2%, whereas all approached controls agreed to participate.

### 2.2. Data Collection

Information was collected using a structured, predesigned questionnaire [16–18]. All the information was self‐reported by the study participants. The interviewers asked questions related to the participant′s lifetime residential history, reproductive history, personal medical history, family history of cancer, tobacco and alcohol consumption, exposure to second‐hand smoking, and basic demographic information.

Reproductive history included information on age at menarche, and detailed history of all pregnancies (including age at pregnancy and breastfeeding information). Personal medical history included information regarding history of common diseases as well as history of benign breast lump. Information on exposure of second‐hand smoking was also collected by asking study participants about their exposure to second‐hand tobacco smoke both at home (if any family member smoked) and outside the home (if any co‐worker or friend smoked). Details such as the type of tobacco product, age at the start of exposure and age at cessation of exposure were recorded. Thorough information was collected regarding participants′ tobacco consumption habits (Supplementary figure 2). This included the use of both smoking and chewing tobacco products. Any individual who consumed tobacco (smoking or chewing) at least once in a week for 6 months was considered a tobacco user and specific details about their consumption pattern were recorded. For chewing tobacco, questions on starting and age at stopping of the use were asked. The questionnaire contained names of specific chewing tobacco products (gutka, mawa, zarda, dohra etc.) divided into broad categories of tobacco, tobacco products with areca nut, tobacco products without areca nut, tobacco for application (like gul, mishri and tapkeer), tobacco for gargling and other products with tobacco. Hormone receptor status of BC cases was abstracted from the electronic medical record. The detailed procedure for ascertainment for hormone receptor status followed by the hospital is available in Appendix 1. For the estimation of the population attributable fraction (PAF), we collected the smokeless tobacco prevalence data (in women) from the Global Adult Tobacco Survey (GATS‐2) India (2016–2017) [19].

### 2.3. Quality Control

All the interviewers across the different hospitals included in the study were trained at the same place (Centre for Cancer Epidemiology, TMC, Mumbai), using the same set of guidelines, to ensure homogeneity in the data collected. They were also given refresher training at regular intervals. All the responses recorded were checked for any implausible responses and missing information. In order to ensure accurate data entry, double data entry was done. Few study participants were reinterviewed and were asked about immutable factors such as age, reproductive history and tobacco consumption, to assess the reliability of the data collected. A shortened version of the original questionnaire was used for this purpose.

This study was approved by the Institutional Ethics Committee of the Tata Memorial Hospital, Mumbai (Approval ID: 3302). The research was conducted in accordance with the Declaration of Helsinki.

### 2.4. Statistical Analysis

The final analysis was performed on 2553 histopathologically confirmed BC cases, and 2239 controls. All the participants included in the analysis were nonsmokers. To summarize selected characteristics of cases and controls, descriptive statistics were performed; *t*‐test was used to compare means between groups for continuous variables, while chi‐square test was used for categorical variables. To assess the association between tobacco chewing as well as other related factors such as duration of tobacco chewing, age at initiation, and duration of use defined by reproductive event with the risk of BC, we used unconditional logistic regression model [20]. Unconditional logistic regression model [20] was used to calculate odds ratio (OR) and its 95% confidence interval (CI). The model was adjusted for important confounders like age (continuous), current residential region (north/south/west/east/central), education (< 5 years and/ ≥ 5 years), age at menarche (continuous), parity (continuous), age at first full‐term pregnancy (continuous), BMI (continuous), history of breastfeeding (yes/no), history of benign breast lump (yes/no), history of BC in first degree relatives (yes/no) and duration of exposure to passive smoking (continuous). The duration of exposure to second‐hand smoking was calculated based on the reported ages of exposure onset and age at stop, with summation performed for multiple exposure periods. For participants with multiple exposure intervals that overlapped, overlapping years were counted only once to avoid duplication. This calculation was performed using SQL Server 2019 [21]. The reference category for all the analysis consisted of women who never consumed tobacco in any form (smoking or chewing). For evaluating the association of duration of tobacco chewing and age at initiation of tobacco chewing, with BC risk, we created tertile for duration and age at initiation of chewing tobacco (based on cut‐off values from controls). We also created categories: after first full‐term pregnancy (FFTP), before FFTP, ≤ 5 years before FFTP, and > 5 years before FFTP, to assess the effect of reproductive events on risk of BC linked initiation of tobacco chewing. The *p* value for increasing trend for OR in each category of duration of chewing and age at initiation was estimated by using the categorical variable (three categories) as continuous variable in the logistic regression model. The analysis was stratified for menopausal status: premenopausal and postmenopausal, molecular subtypes of BC (based on their hormone receptor status): ER and/or PR+, HER2−, ER and/or PR+, HER2+, triple negative breast cancer (TNBC) and BMI: underweight: ≤18.5 kg/m^2^, normal:18.5–24.9 kg/m^2^, overweight: 24.9–30 kg/m^2^, obese: > 30 kg/m^2^ (as per the World Health Organization classification) [22]. A significance level of *p*‐value ≤ 0.05 was used to determine statistical significance throughout the analyses.

We also calculated the attributable fraction (AF) of BC due to chewing tobacco amongst the cases in our study population. For the AF calculation we used the Miettinen′s formula [23]. The prevalence of chewing tobacco was estimated from our study population (in BC cases only). State‐wise PAF of BC due to chewing tobacco was also estimated, using Levin′s formula [23]. The prevalence data was derived from the general population (GATS‐2), assuming a causal relationship between tobacco chewing and BC [24–26]. The OR used in the calculation of both AF and PAF was derived from the current study. All analyses were performed on Stata Version 15 [27].

## 3. Results

Baseline characteristics for study participants are presented in Table 1. The cases were slightly older than the controls (~48 vs. ~47 years). The mean age at menarche was not significantly different for cases and controls; however, the mean age at FFTP was slightly higher in cases (~23 years) as compared with controls (~22 years). A higher proportion of cases were postmenopausal women (55%), whereas in controls, the majority of women were premenopausal (~53%). No significant difference between cases and controls was observed with regard to history of breastfeeding and BMI. More proportion of cases than controls had a history of benign breast lump (9% vs. 5%). While the distribution of cases and controls based on demographic factors such as current residential zone and education differed slightly, the majority of cases and controls came from the western region of the country (> 55%) and had more than or equal to 5 years of education (> 70%).

**Table 1:** Summary characteristics of the study population for selected variables.

**Variable name**	**Cases (%)** **(N=2553)**	**Controls (%)** **(N=2239)**	**p** **value**
Age (completed age at enrolment), mean (±SD)	48.20 (±7.87)	46.84 (±7.73)	**≤0.001**
Age at menarche, mean (±SD)	13.76 (±1.51)	13.82 (±1.55)	0.219
Age at first full term pregnancy, mean (±SD)	22.72 (±4.60)	22.22 (±4.59)	**≤0.001**
Parity, mean (±SD)	2.69 (±1.34)	2.68 (±1.31)	0.913
Menopausal status			
Premenopausal	1148 (44.97)	1179 (52.66)	**≤0.001**
Postmenopausal	1405 (55.03)	1060 (47.34)	
Missing	0 (0.00)	0 (0.00)	
History of Breastfeeding			
Yes	2335 (99.11)	2007 (99.01)	0.744
No	21 (0.89)	20 (0.99)	
Missing	0 (0.00)	0 (0.00)	
History of benign breast lump			
Yes	233 (9.13)	106 (4.73)	**≤0.001**
No	2313 (90.60)	2127 (95.00)	
Missing	7 (0.27)	6 (0.27)	
BMI (in kg/m^2^)			
Underweight (<16.5‐18.5)	133 (5.21)	91 (4.06)	0.096
Normal (18.5‐24.9)	1029 (40.31)	863 (38.54)	
Overweight (24.9‐30)	961 (37.64)	876 (39.12)	
Obesity (>30)	430 (16.84)	409 (18.27)	
Missing	0 (0.00)	0 (0.00)	
Current region of residence (at the time of enrolment)			
Central	120 (4.70)	91 (4.06)	**0.005**
East	464 (18.17)	462 (20.63)	
North	540 (21.15)	391 (17.46)	
South	22 (0.86)	26 (1.16)	
West	1407 (55.11)	1269 (56.68)	
Missing	0 (0.00)	0 (0.00)	
Education			
Less than five years schooling	760 (29.77)	490 (21.88)	**≤0.001**
Five or more years schooling	1793 (70.23)	1749 (78.12)	
Missing	0 (0.00)	0 (0.00)	
Family history of breast cancer in first degree relatives			
Yes	95 (3.72)	26 (1.16)	**≤0.001**
No	2442 (95.65)	2190 (97.81)	
Missing	16 (0.63)	23 (1.03)	
Duration of exposure to second‐hand smoking, mean (±SD)	19.78 (±11.02)	20.17 (±11.77)	0.569

Abbreviations: n, number; SD, standard deviation.

Numbers are presented as n (%) unless otherwise stated.

Significant p‐values are presented in boldface.

The mean age of cases and controls differed significantly only among ER and/or PR‐positive, HER2‐negative cases and ER and/or PR‐positive, HER2‐positive cases, but not among those with triple‐negative BC. The mean age at first full‐term pregnancy and the mean parity differed only between cases and controls with ER and/or PR‐positive, HER2‐negative BC. In contrast, the distribution of participants by menopausal status, history of benign breast lump, current residential zone, education level and family history of breast cancer differed across all molecular subtypes of BC (Table S2). Table 2 shows the association between chewing tobacco in all study participants as well as different molecular subtypes of BC. An increased risk of BC was observed in women who ever used chewing tobacco (OR: 1.19, 95% CI:1.00–1.41) as compared with those who never consumed tobacco (smoking and chewing) in their lifetime. This was observed across molecular subtypes of BC, with highest risk for ER and/or PR+, HER2+ subtype (OR: 1.58, 95% CI: 1.16–2.16). Dose‐response relation was observed for duration of tobacco chewing with *p* value for trend (for all study participants) of 0.017 (OR > 25 years: 1.38, 95% CI: 1.04–1.83). Higher duration of chewing tobacco (> 25 years) also consistently increased the risk of all the molecular subtypes of BC, with OR ranging from 1.28 to 1.80 (although not statistically significant for some molecular subtypes of BC). Women who started chewing tobacco earlier in life (< 20 years of age) had 1.3 times higher chances of developing BC, about 1.8 times higher risk of developing ER and/or PR+, HER2 + BC and triple‐negative BC, as compared with nontobacco users. With regard to the association between BC and starting chewing tobacco before/after FFTP, we observed about 1.4 times risk of BC in women who started chewing tobacco before their FFTP, specifically among those who started chewing tobacco ≤ 5 years before their FFTP (OR≤5 years before their FFTP: 1.65, 95% CI:1.20–2.27, for all study participants). Similar trend was observed for risk of developing specific molecular subtypes of BC.

**Table 2:** Association of tobacco chewing with the risk of molecular subtypes of breast cancer.

**Variables**	**All study participants** **(Cases=2553, Controls=2239)**			**ER and/or PR +, HER2 -** **(Cases=867, Controls=2239)**			**ER and/or PR +, HER2 +** **(Cases=365, Controls=2239)**			**Triple Negative Breast Cancer** **(Cases=451, Controls=2239)**		
	**Case/control**	**OR (95% CI)**	**p** **value**	**Case/control**	**OR (95% CI)**	**p** **value**	**Case/control**	**OR (95% CI)**	**p** **value**	**Case/control**	**OR (95% CI)**	**p** **value**
Chewing tobacco												
Non‐tobacco users	2125/1943	Ref		745/1943	Ref		291/1943	Ref		372/1943	Ref	
Ever tobacco chewer	428/296	**1.19 (1.00-1.41)**	0.049	122/296	1.07 (0.83‐1.37)	0.610	74/296	**1.58 (1.16-2.16)**	0.004	79/296	**1.35 (1.01-1.82)**	0.044
Duration of tobacco chewing (in years)												
Non‐tobacco users	2125/1943	Ref		745/1943	Ref		291/1943	Ref		372/1943	Ref	
<11	138/115	1.05 (0.81‐1.37)	0.711	35/115	0.80 (0.52‐1.21)	0.285	24/115	1.41 (0.87‐2.29)	0.165	26/115	1.21 (0.77‐1.91)	0.417
11‐25	120/87	1.18 (0.88‐1.58)	0.279	36/87	1.20 (0.79‐1.84)	0.390	20/87	1.57 (0.93‐2.66)	0.093	22/87	1.35 (0.82‐2.23)	0.234
>25	170/94	**1.38 (1.04-1.83)**	0.026	51/94	1.28 (0.87‐1.90)	0.215	30/94	**1.80 (1.11-2.92)**	0.018	31/94	1.54 (0.96‐2.47)	0.071
P_trend_	0.017			0.234			0.003			0.031		
Age at initiation of chewing tobacco (in years)												
Non‐tobacco users	2125/1943	Ref		745/1943	Ref		291/1943			372/1943	Ref	
<20	167/101	**1.35 (1.03-1.78)**	0.031	54/101	1.41 (0.96‐2.05)	0.078	28/101	**1.79 (1.11-2.88)**	0.017	37/101	**1.81 (1.18-2.77)**	0.006
20‐30	142/99	1.22 (0.93‐1.61)	0.157	42/99	1.11 (0.75‐1.64)	0.615	26/99	**1.73 (1.08-2.77)**	0.023	18/99	0.97 (0.57‐1.65)	0.904
>30	119/96	0.99 (0.74‐1.33)	0.978	26/96	0.70 (0.44‐1.12)	0.136	20/96	1.22 (0.70‐2.11)	0.487	24/96	1.29 (0.79‐2.09)	0.308
P_trend_	0.257			0.601				0.033			0.198	
Initiation of chewing tobacco defined by reproductive event (only for those ever got pregnant)												
Non‐tobacco users	1968/1764	Ref		731/1764	Ref		285/1764	Ref		363/1764	Ref	
After first full‐term pregnancy	181/140	1.12 (0.88‐1.42)	0.369	49/140	0.88 (0.62‐1.26)	0.485	33/140	**1.51 (1.00-2.30)**	0.052	32/140	1.08 (0.70‐1.64)	0.733
Before first full‐term pregnancy	207/123	**1.41 (1.10-1.80)**	0.006	66/123	1.30 (0.93‐1.81)	0.126	35/123	**1.71 (1.12-2.60)**	0.013	44/123	**1.68 (1.15-2.46)**	0.007
≤5 years before first full‐term pregnancy	127/67	**1.65 (1.20-2.27)**	0.002	43/67	**1.63 (1.07-2.49)**	0.023	22/67	**2.06 (1.21-3.49)**	0.008	25/67	**1.87 (1.15-3.05)**	0.012
>5 years before first full‐term pregnancy	80/56	1.13 (0.79‐1.62)	0.496	23/56	0.95 (0.57‐1.58)	0.845	13/56	1.33 (0.70‐2.51)	0.378	19/56	1.47 (0.84‐2.57)	0.176

Abbreviations: OR, Odds Ratio; CI, Confidence Interval.

Odds ratio with significant p‐values have been presented in boldface.

All odds ratio have been adjusted for Age (continuous), current residential region (North/South/West/East/Central), education (<5 years / ≥5 years), age at menarche (continuous), parity (continuous), age at first full‐term pregnancy (continuous), Menopausal status (premenopausal/postmenopausal), BMI (continuous), benign breast lump (yes/no), history of breastfeeding (yes/no), family history of breast cancer in first degree relatives (yes/no) and duration of exposure to second‐hand smoking (in years, continuous).

The positive association between chewing tobacco and the risk of developing BC was consistent even after stratifying on the basis of menopausal status (Table 3), although statistically not significant (OR premenopausal: 1.20, 95% CI: 0.90–1.59; OR postmenopausal: 1.17, 95% CI: 0.94–1.46). In both premenopausal and postmenopausal women, dose response with duration of chewing tobacco was observed. Women who started chewing tobacco earlier had higher risk of BC (OR < 20‐years, postmenopausal: 1.47, 95% CI: 1.04–2.09) amongst the postmenopausal women. This was not replicated in premenopausal women. With respect to initiation of chewing tobacco defined by reproductive event, results were consistent for premenopausal (statistically nonsignificant), as well as postmenopausal women. Stratifying individuals based on their BMI did not reveal any differences in the ORs across the BMI strata (Table S3).

**Table 3:** Association of tobacco chewing with the risk of breast cancer.

**Variables**	**Premenopausal** **(Cases=1148, controls=1179)**			**Postmenopausal** **(Cases=1405, controls=1060)**		
	**Case/control**	**OR (95% CI)**	**p** **value**	**Case/control**	**OR (95% CI)**	**p** **value**
Chewing tobacco						
Non‐tobacco users	1019/1062	Ref		1106/881	Ref	
Ever tobacco chewer	129/117	1.20 (0.90‐1.59)	0.205	299/179	1.17 (0.94‐1.46)	0.155
Duration of tobacco chewing (in years)						
Non‐tobacco users	1019/1062	Ref		1106/881	Ref	
<11	53/51	1.12 (0.74‐1.69)	0.593	85/64	1.00 (0.70‐1.42)	0.998
11‐25	50/48	1.16 (0.76‐1.77)	0.489	70/39	1.23 (0.81‐1.87)	0.337
>25	26/18	1.56 (0.81‐2.98)	0.18	144/76	1.30 (0.95‐1.78)	0.105
P_trend_	0.142			0.078		
Age at initiation of chewing tobacco (in years)						
Non‐tobacco users	1019/1062			1106/881	Ref	
<20	45/44	1.15 (0.73‐1.80)	0.553	122/57	**1.47 (1.04-2.09)**	0.031
20‐30	49/49	1.09 (0.71‐1.66)	0.695	93/50	1.34 (0.93‐1.95)	0.119
>30	35/24	1.52 (0.88‐2.63)	0.137	84/72	0.83 (0.59‐1.17)	0.293
P_trend_	0.149			0.798		
						
Non‐tobacco users	952/946	Ref		1016/818	Ref	
After first full‐term pregnancy	57/55	1.08 (0.72‐1.61)	0.718	124/85	1.13 (0.83‐1.53)	0.44
Before first full‐term pregnancy	64/51	1.41 (0.95‐2.10)	0.085	143/72	**1.40 (1.02-1.91)**	0.036
≤5 years before first full‐term pregnancy	42/32	1.53 (0.94‐2.49)	0.085	85/35	**1.72 (1.13-2.63)**	0.012
>5 years before first full‐term pregnancy	22/19	1.22 (0.64‐2.32)	0.537	58/37	1.10 (0.71‐1.71)	0.655

Abbreviations: OR, Odds Ratio; CI, Confidence Interval.

Odds ratio with significant p‐values have been presented in boldface.

All odds ratio have been adjusted for Age (continuous), current residential region (North/South/West/East/Central), education (<5 years / ≥5 years), age at menarche (continuous), parity (continuous), age at first full‐term pregnancy (continuous), BMI (continuous), benign breast lump (yes/no), history of breastfeeding (yes/no), family history of breast cancer in first degree relatives (yes/no) and duration of exposure to second‐hand smoking (in years, continuous).

The AF of BC due to tobacco chewing in our study population was calculated to be about 3% (Table S4), whereas PAF for India was calculated to be about 2% (Table S5). The top three states with highest PAF of BC due to tobacco chewing were Tripura (9.69%), Mizoram (8.04%) and Manipur (7.91%), on the other hand Himachal Pradesh (0.02%), Punjab (0.06%) and Chandigarh (0.15%) had the lowest PAF (Table S5).

## 4. Discussion

In the current study, we observed a significant positive association between chewing tobacco and the risk of BC, with slightly stronger associations for certain molecular subtypes and with earlier initiation and longer duration of use. Increased odds of BC were similar for both premenopausal and postmenopausal women. The association of BC with chewing tobacco was uniform across all BMI categories, indicating no confounding effect of BMI on the observed risk. These findings highlight the potential role of smokeless tobacco specifically chewing tobacco as an important, yet underrecognized modifiable risk factor for BC, particularly in the Indian population where its use is prevalent among women. We additionally found that the AF of BC associated with tobacco chewing was approximately 3% amongst the cases in our study population, whereas the corresponding PAF for India as a whole was about 2%.

The observed association between BC and chewing tobacco is biologically plausible. Chewing tobacco products contain multitude of harmful chemicals such as tobacco specific nitrosamines (TSNAs), polyaromatic hydrocarbons, inorganic metals and salts [24]. These are metabolized in human body by cytochrome P450 enzymes, particularly CYP1A1 and CYP1B1, this metabolic process leads to the formation of DNA adducts, causing mutations in critical tumour suppressor genes like TP53, which has previously been reported to influence initiation of BC development [25]. Additionally, some chemicals in chewing tobacco, including nicotine, and NNK, promote inflammation by increasing levels of interleukin‐6 and tumour necrosis factor‐alpha. These inflammatory molecules activate signalling pathways that can enhance the activity of aromatase, the enzyme responsible for converting androgens into estrogens (Figure S3). The elevated estrogen levels in breast tissue can in turn stimulate the growth of estrogen receptor‐positive (ER^+^) cancer cells [28]. Furthermore, nicotine and TSNAs directly activate estrogen receptors (ER*α* and ER*β*) without binding and promoting the expression of genes like c‐Myc and cyclin D1 that drive cell proliferation in the breast [26]. Concurrently, CYP1B1‐mediated conversion of estrogen into reactive catechol metabolites generates reactive oxygen species (ROS), resulting in further DNA damage and mutations [28]. Together, these biological mechanisms may cause BC through DNA damage, hormone disruption, and chronic inflammation, induced by SLT. Initiating the use of chewing tobacco before the FFTP increased the risk of BC about 1.4 times, specifically if it was initiated ≤ 5 years before FFTP. One meta‐analyses by He et al. showed that smoking before the first birth had greater impact on risk of BC than smoking after the first birth [9]. It is argued that the breast tissues are particularly vulnerable to the carcinogenic effect of smoking before first pregnancy since it is undifferentiated [29, 30]. This has been proven in animal models, where it was observed that cancer initiation can occur when chemical carcinogens come into contact with undifferentiated, highly proliferating mammary epithelium. On the other hand, cancer initiation is unlikely after the first pregnancy that triggers breast growth and mammary gland differentiation [29].

Only a few studies have reported increased risk of BC with consumption of SLT [13, 14, 31], and increase in risk as duration of use increases [15, 31]. As existing evidence suggest the link between SLT use with various cancers, including oral [32], oesophageal and pancreatic cancers [33], our study adds to a growing body of evidence suggesting its role in breast carcinogenesis. The results of the current study are strengthened by the dose‐response relationship observed between duration of tobacco use and the risk of BC, which has previously been reported by other studies [15, 31]. However, research on the association between SLT (more specifically chewing tobacco) and BC still remains limited, particularly in low‐ and middle‐income countries where its use is more prevalent among women.

To the best of our knowledge, this is the first large‐scale case‐control study from India that has studied the role of chewing tobacco in the risk of developing BC. The study population consists of women from different regions of India, capturing diverse exposures and tobacco habits. Our study had enough power to estimate true odds ratio for BC (Figure S4). We collected detailed information on tobacco use pattern and important confounding variables such as current residential region, education, age at menarche, parity, age at FFTP, exposure to second‐hand smoking, family history of BC and so on. Moreover, our study population did not have any smokers, thereby completely removing the confounding effect of smoking on the risk of BC and providing risk estimates solely due to chewing tobacco. Additionally, alcohol consumption is uncommon among Indian women; in our study, less than 1% reported drinking alcohol. As a result, the potential confounding effect of alcohol—often seen in studies from Western populations is also negligible in our analysis. However, some limitations should be acknowledged. Although it is unlikely that an individual has difficulty recalling their tobacco consumption habits, the reliance on self‐reported data may introduce recall bias, particularly in retrospective assessment of tobacco use history. However, when we reinterviewed a sample of the study participants and asked them regarding their tobacco consumption history, we observed high agreement rate (> 94%) for initial response and response during the re‐interview. Due to case‐control study design, our study results may also be subject to selection bias. Even though it is impossible to completely eliminate such bias, we attempted to minimize it as much as possible by ensuring that our cases and controls come from the same source population. Moreover, we cannot establish temporality for the association observed, since this is a retrospective study. Despite our attempt to control for key sociodemographic and reproductive factors, residual confounding due to unmeasured confounders cannot be entirely ruled out. Additionally, even though the prevalence used for the PAF calculation in this study was obtained from the general population (GATS‐2), the case–control design of our study implies that the generalisability of our findings to the general population may be limited. However, there is a biological plausibility for the association observed and the odds ratio used for AF, and PAF calculations was adjusted for key confounders, strengthening the validity of the causal inference. Despite these limitations, our results underscore the need for increased public health awareness regarding the risks of smokeless tobacco in Indian women.

## 5. Conclusion

In conclusion, our study suggests that chewing tobacco use is associated with an increased risk of BC, for all molecular subtypes, particularly when the duration of exposure was higher and when exposure begins before FFTP. These findings warrant further prospective studies and mechanistic research to better understand the biological pathways involved. Importantly, they highlight the need to address SLT use as part of comprehensive cancer prevention strategies, particularly in regions such as east of India, where its use is widespread among women and PAF of BC due to tobacco chewing is also high. Tobacco control policies should not only target smoking but also address SLT use, which is culturally accepted, to reduce the burden of BC to some extent, specifically in parts of India, where the prevalence of other risk factors of BC is less prevalent.

## Disclosure

R.M., A.M., G.B.P., S.V.G., R.S.M., S.S., D.T., N.C., R.P., S.P., M.K., N.P., R.D., I.S., S.G. and S.M. approved the final version of the manuscript. All the authors are accountable for all aspects of the work.

## Conflicts of Interest

The authors declare no conflicts of interest.

## Author Contributions

R.D. and S.M. were involved in the conception, design and direction of the study. N.P., R.P., M.K., A.M., and S.S. supervised the fieldwork. A.M., S.S. and S.M. undertook training of the staff. A.M., S.S. and S.M. monitored data collection and management. S.M., S.V.G., A.M., D.T., N.C. and S.S. were involved in collection and assembly of data. Analysis was conducted by R.M. and G.B.P., and was directed by R.D. and S.M. R.M., A.M., G.B.P., S.V.G., R.S.M., S.S., D.T., N.C., R.P., S.P., M.K., N.P., R.D., I.S., S.G. and S.M. were involved in interpretation of results. R.M. led in writing the manuscript. Subsequent drafts were revised by all authors.

## Funding

This study was supported by theTata Memorial Centre (10.13039/501100010476), intramural fund

## Supporting information


**Supporting Information 1** Additional supporting information can be found online in the Supporting Information section. Figure S1: Inclusion and exclusion criteria for cases and controls. Table S1: Distribution of controls in various DMGs. Figure S2: Image showing ‘chewing tobacco section’ in the case‐control study. Table S2: Summary characteristics of the study population for selected variables, stratified by hormone receptor status. Table S3: Association of tobacco chewing with the risk of breast cancer for all study participants stratified by BMI. Table S4: Attributable fraction calculation. Table S5: Population attributable fraction calculation. Figure S3: Flowchart showing how nicotine and NNK trigger inflammation and activate PI3K/Akt and MAPK pathways. Figure S4: Graphical representation of sample size required to estimate different levels of odds ratio.

## Data Availability

The datasets used and/or analysed during the current study are available from the corresponding author on reasonable request.

## Appendix 1 Methodology for Assessing Hormonal Receptor Status in Breast Cancer Tissue Using Immunohistochemistry (IHC)

Biomarker assessment for estrogen receptor (ER) (SP1, Ventana Medical System Inc, Tucson, AZ), progesterone receptor (PR) (1E2, Ventana Medical System Inc) and human epidermal growth factor receptor 2 (HER2) (4B5, Ventana Medical System Inc) was performed on Ventana BenchMark automated immunostainer. Formalin‐fixed, paraffin‐embedded (FFPE) biopsy or surgical resection specimens were used for immunohistochemistry, and the results were evaluated according to the American Society of Clinical Oncology/College of American Pathologists (ASCO/CAP) guidelines [1, 2].

Stained slides were assessed by a pathologist. ER and PR expression were interpreted based on the percentage of positively stained tumour nuclei and staining intensity. Tumours were classified as hormone receptor–positive if ER and/or PR expression was ≥1% with an Allred score ≥3. HER2/neu scores of 0 or 1+ were considered HER2‐negative, while 3+ was considered HER2‐positive. Equivocal cases (2+) underwent in situ hybridisation (ISH) using fluorescence ISH (FISH) with the ZytoLight® SPEC ERBB2/CEN17 Dual Colour Probe Kit (ZytoVision GmbH, Bremerhaven, Germany). ISH results were classified as HER2‐amplified (HER2‐positive) or non‐amplified (HER2‐negative).

**Table:**

**Status**	**Criteria**
Hormone receptor positive	➢ ER and/or PR expression was ≥1% and ➢ Allred score ≥3
HER2/neu negative	Scores of 0 or 1+
HER2/neu positive	Score of 3+
HER2/neu equivocal	Score of 2+; suggested for FISH ➢ If HER2‐amplified then HER2‐positive ➢ If HER2 non‐amplified then HER2‐negative.

Cases were categorised into surrogate molecular subtypes as follows: hormone receptor–positive/HER2–, triple‐positive (hormone receptor–positive/HER2+), and triple‐negative breast cancer (TNBC; hormone receptor–negative/HER2–).

### References for Appendix

1. Wolff AC, Hammond MEH, Allison KH, Harvey BE, Mangu PB, Bartlett JMS, et al. Human Epidermal Growth Factor Receptor 2 Testing in Breast Cancer: American Society of Clinical Oncology/College of American Pathologists Clinical Practice Guideline Focused Update. J Clin Oncol Off J Am Soc Clin Oncol. 2018 July 10;36(20):2105–22.
2. Hammond MEH, Hayes DF, Dowsett M, Allred DC, Hagerty KL, Badve S, et al. American Society of Clinical Oncology/College Of American Pathologists guideline recommendations for immunohistochemical testing of estrogen and progesterone receptors in breast cancer. J Clin Oncol Off J Am Soc Clin Oncol. 2010 June 1;28(16):2784–95.
