# Supplementary material for: Association of Chewing Tobacco and the Risk of Breast Cancer in Indian Women: A Multicentre Case‐Control Study
Source: Int J Breast Cancer. 2026 Mar 9;2026:2950851. doi: 10.1155/ijbc/2950851 (PMC12969211; doi:10.1155/ijbc/2950851)
Supplement: Supplementary file 1 — Supporting Information 1 Additional supporting information can be found online in the Supporting Information section. Figure S1: Inclusion and exclusion criteria for cases and controls. Table S1: Distribution of controls in various DMGs. Figure S2: Image showing ‘chewing tobacco section’ in the case‐control study. Table S2: Summary characteristics of the study population for selected variables, stratified by hormone receptor status. Table S3: Association of tobacco chewing with the risk of breast cancer for all study participants stratified by BMI. Table S4: Attributable fraction calculation. Table S5: Population attributable fraction calculation. Figure S3: Flowchart showing how nicotine and NNK trigger inflammation and activate PI3K/Akt and MAPK pathways. Figure S4: Graphical representation of sample size required to estimate different levels of odds ratio. [file IJBC-2026-2950851-s001.docx]

**Supplementary Materials**

***
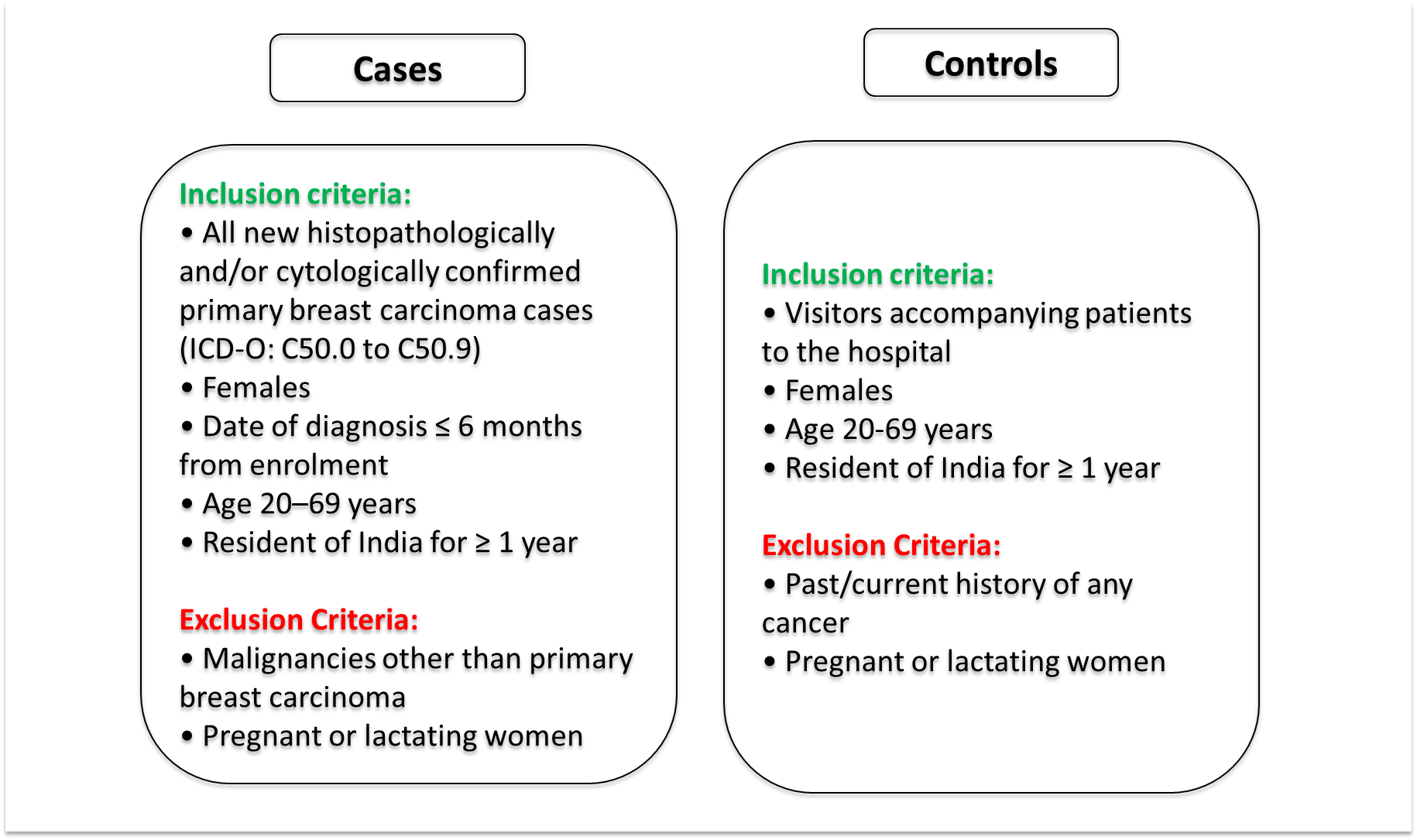
Supplementary figure 1***: Inclusion and exclusion criteria for cases and controls

| ***Supplementary table 1:*** Distribution of controls in various DMGs | |
| --- | --- |
| **Disease Management Group (DMG)^#^** | **Number (%)** |
| Adult haematolymphoid | 184 (8.22) |
| Bone and soft tissue | 152 (6.79) |
| Breast | 30 (1.34) |
| Gastrointestinal | 538 (24.03) |
| Gynaecology | 87 (3.89) |
| Head and Neck | 679 (30.33) |
| Neuro Oncology | 69 (3.08) |
| Paediatric haemato lymphoid | 83 (3.71) |
| Paediatric solid tumours | 31 (1.38) |
| Preventive oncology | 10 (0.45) |
| Thoracic | 209 (9.33) |
| Urology | 161 (7.19) |
| Missing | 6 (0.27) |
|  |  |
| *Note: DMG is of the patient whom the control was accompanying.  ^#^DMGs are units that specialise in treating specific cancer sites in the hospital (e.g. Breast cancer cases are treated under the Breast DMG) | |

***Supplementary figure 2****:* Image showing “chewing tobacco section” in the case-control study


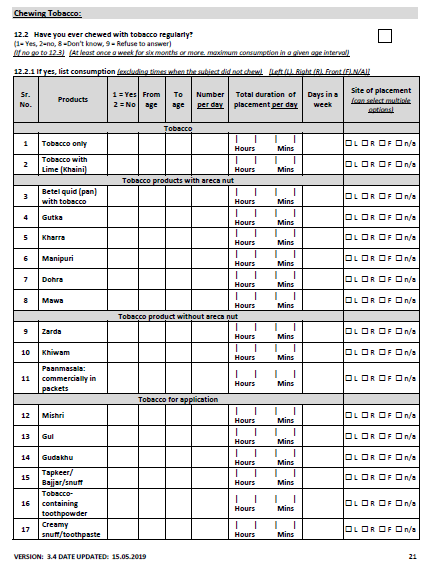


| ***Supplementary table 2:*** Summary characteristics of the study population for selected variables, stratified by hormone receptor status | | | | | | | | | |
| --- | --- | --- | --- | --- | --- | --- | --- | --- | --- |
|  | **ER and/or PR +, HER2 -** | | | **ER and/or PR +, HER2 +** | | | **Triple Negative Breast Cancer** | | |
| **Variable name** | **Cases (%)** (N1=867) | **Controls (%)** (N2=2239) | **p- value** | **Cases (%)** (N3=365) | **Controls (%)** (N2=2239) | **p- value** | **Cases (%)** (N4=451) | **Controls (%)** (N2=2239) | **p- value** |
| Age (completed age at enrolment), mean (±SD) | 48.62 (±7.80) | 46.84 (±7.73) | **≤0.001** | 47.77 (±7.65) | 46.84 (±7.73) | **0.033** | 47.09 (±7.72) | 46.85 (±7.73) | 0.526 |
| Age at menarche, mean (±SD) | 13.79 (±1.53) | 13.82 (±1.55) | 0.622 | 13.78 (±1.52) | 13.82 (±1.55) | 0.645 | 13.78 (±1.57) | 13.82 (±1.55) | 0.598 |
| Age at first full term pregnancy, mean (±SD) | 23.38 (±4.57) | 22.22 (±4.59) | **≤0.001** | 22.64 (±4.27) | 22.22 (±4.59) | 0.1043 | 22.63 (±4.49) | 22.22 (±4.59) | 0.089 |
| Parity, mean (±SD) | 2.52 (±1.19) | 2.68 (±1.31) | **0.001** | 2.69 (±1.33) | 2.68 (±1.31) | 0.950 | 2.73 (±1.37) | 2.68 (±1.31) | 0.513 |
| Menopausal status |  |  |  |  |  |  |  |  |  |
| Premenopausal | 400 (46.14) | 1179 (52.66) | **≤0.001** | 170 (46.58) | 1179 (52.66) | **≤0.001** | 221 (49.00) | 1179 (52.66) | **≤0.001** |
| Postmenopausal | 467 (53.86) | 1060 (47.34) |  | 195 (53.42) | 1060 (47.34) |  | 230 (51.00) | 1060 (47.34) |  |
| Missing | 0 (0.00) | 0 (0.00) |  | 0 (0.00) | 0 (0.00) |  | 0 (0.00) | 0 (0.00) |  |
| History of Breastfeeding |  |  |  |  |  |  |  |  |  |
| Yes | 817 (98.67) | 2007 (99.01) | 0.254 | 349 (98.87) | 2007 (99.01) | 0.832 | 437 (99.54) | 2007 (99.01) | 0.545 |
| No | 11 (1.33) | 20 (0.99) |  | 4 (1.13) | 20 (0.99) |  | 2 (0.46) | 20 (0.99) |  |
| Missing | 0 (0.00) | 0 (0.00) |  | 0 (0.00) | 0 (0.00) |  | 0 (0.00) | 0 (0.00) |  |
| History of benign breast lump |  |  |  |  |  |  |  |  |  |
| Yes | 89 (10.27) | 106 (4.73) | **≤0.001** | 37 (10.14) | 106 (4.73) | **≤0.001** | 25 (5.54) | 106 (4.73) | **≤0.001** |
| No | 778 (89.73) | 2127 (95.00) |  | 323 (88.49) | 2127 (95.00) |  | 425 (94.24) | 2127 (95.00) |  |
| Missing | 0 (0.00) | 6 (0.27) |  | 5 (1.37) | 6 (0.27) |  | 1 (0.22) | 6 (0.27) |  |
| BMI (in kg/m^2^) |  |  |  |  |  |  |  |  |  |
| Underweight (<16.5-18.5) | 30 (3.46) | 91 (4.06) | **0.001** | 18 (4.93) | 91 (4.06) | 0.346 | 25 (5.54) | 91 (4.06) | 0.322 |
| Normal (18.5-24.9) | 334 (38.52) | 863 (38.54) |  | 143 (39.18) | 863 (38.54) |  | 186 (41.24) | 863 (38.54) |  |
| Overweight (24.9-30) | 331 (38.18) | 876 (39.12) |  | 140 (38.36) | 876 (39.12) |  | 169 (37.47) | 876 (39.12) |  |
| Obesity (>30) | 172 (19.84) | 409 (18.27) |  | 64 (17.53) | 409 (18.27) |  | 71 (15.74) | 409 (18.27) |  |
| Missing | 0 (0.00) | 0 (0.00) |  | 0 (0.00) | 0 (0.00) |  | 0 (0.00) | 0 (0.00) |  |
| Current region of residence (at the time of enrolment) |  |  |  |  |  |  |  |  |  |
| Central | 48 (5.54) | 91 (4.06) | **0.002** | 23 (6.30) | 91 (4.06) | **0.025** | 21 (4.66) | 91 (4.06) | **0.017** |
| East | 171 (19.72) | 462 (20.63) |  | 65 (17.81) | 462 (20.63) |  | 92 (20.40) | 462 (20.63) |  |
| North | 164 (18.92) | 391 (17.46) |  | 79 (21.64) | 391 (17.46) |  | 94 (20.84) | 391 (17.46) |  |
| South | 11 (1.27) | 26 (1.16) |  | 3 (0.82) | 26 (1.16) |  | 1 (0.22) | 26 (1.16) |  |
| West | 473 (54.56) | 1269 (56.68) |  | 195 (53.42) | 1269 (56.68) |  | 243 (53.88) | 1269 (56.68) |  |
| Missing | 0 (0.00) | 0 (0.00) |  | 0 (0.00) | 0 (0.00) |  | 0 (0.00) | 0 (0.00) |  |
| Education |  |  |  |  |  |  |  |  |  |
| Less than five years schooling | 184 (21.22) | 490 (21.88) | **≤0.001** | 119 (32.60) | 490 (21.88) | **≤0.001** | 121 (26.83) | 490 (21.88) | **≤0.001** |
| Five or more years schooling | 683 (78.78) | 1749 (78.12) |  | 246 (67.40) | 1749 (78.12) |  | 330 (73.17) | 1749 (78.12) |  |
| Missing |  |  |  |  |  |  |  |  |  |
| Family history of breast cancer in first degree relatives |  |  |  |  |  |  |  |  |  |
| Yes | 39 (4.50) | 26 (1.16) | **≤0.001** | 10 (2.74) | 26 (1.16) | **≤0.001** | 20 (4.43) | 26 (1.16) | **≤0.001** |
| No | 819 (94.46) | 2190 (97.81) |  | 353(96.71) | 2190 (97.81) |  | 430 (95.34) | 2190 (97.81) |  |
| Missing | 9 (1.04) | 23 (1.03) |  | 2 (0.55) | 23 (1.03) |  | 1 (0.22) | 23 (1.03) |  |
| Duration of exposure to second-hand smoking, mean (±SD) | 20.68 (±10.70) | 20.17 (±11.77) | 0.593 | 19.54 (±11.78) | 20.17 (±11.77) | 0.668 | 18.80 (±10.14) | 20.17 (± 11.77) | 0.272 |
|  |  |  |  |  |  |  |  |  |  |
| *Abbreviations: n, number; SD, standard deviation.  Numbers are presented as n (%) unless otherwise stated.  Significant p-values are presented in boldface.* | | | | | | | | | |

| **Supplementary Table 3 : Association of tobacco chewing with the risk of breast cancer for all study participants stratified by BMI** | | | | | | | | | | | | |
| --- | --- | --- | --- | --- | --- | --- | --- | --- | --- | --- | --- | --- |
|  | **Underweight** (<18.5 kg/m^2^)  (Cases=133,Controls=91) | | | **Normal** (18.5-24.9 kg/m^2^)  (Cases=1029,Controls=863) | | | **Overweight** (24.9-30 kg/m^2^)  (Cases=961,Controls=876) | | | **Obese**  (>30 kg/m^2^)  (Cases=430,Controls=409) | | |
| **Variables** | **Case/control** | **OR (95% CI)** | **p-value** | **Case/control** | **OR (95% CI)** | **p-value** | **Case/control** | **OR (95% CI)** | **p-value** | **Case/control** | **OR (95% CI)** | **p-value** |
| **Chewing tobacco** |  |  |  |  |  |  |  |  |  |  |  |  |
| Non-tobacco users | 102/67 | Ref |  | 828/735 | Ref |  | 814/777 | Ref |  | 381/364 | Ref |  |
| Ever tobacco chewer | 31/24 | 0.83 (0.40-1.69) | 0.604 | 201/128 | 1.24 (0.95-1.61) | 0.116 | 147/99 | 1.25 (0.93-1.67) | 0.134 | 49/45 | 1.00 (0.63-1.59) | 0.992 |
|  |  |  |  |  |  |  |  |  |  |  |  |  |
| **Duration of tobacco chewing (in years)** |  |  |  |  |  |  |  |  |  |  |  |  |
| Non-tobacco users | 102/67 | Ref |  | 828/735 | Ref |  | 814/777 | Ref |  | 381/364 | Ref |  |
| <11 | 6/8 | 1.05 (0.32-3.51) | 0.933 | 65/50 | 1.07 (0.72-1.61) | 0.736 | 52/38 | 1.21 (0.78-1.89) | 0.393 | 15/19 | 0.71 (0.35-1.47) | 0.361 |
| 11-25 | 11/9 | 0.64 (0.22-1.84) | 0.349 | 56/36 | 1.31 (0.83-2.05) | 0.244 | 40/28 | 1.27 (0.76-2.12) | 0.363 | 13/14 | 0.81 (0.36-1.86) | 0.625 |
| >25 | 14/7 | 0.89 (0.29-2.76) | 0.845 | 80/42 | 1.38 (0.90-2.12) | 0.140 | 55/33 | 1.27 (0.79-2.06) | 0.320 | 21/12 | 1.68 (0.79-3.57) | 0.180 |
| P_trend_ | 0.594 |  |  | 0.073 |  |  | 0.156 |  |  | 0.484 |  |  |
| Abbreviations: OR, Odds Ratio; CI, Confidence Interval.  Odds ratio with significant p-values have been presented in boldface.  All odds ratio have been adjusted for Age (continuous), current residential region (North/South/West/East/Central), education (<5 years / ≥5 years), age at menarche (continuous), parity (continuous), age at first full-term pregnancy (continuous), Menopausal status (premenopausal/postmenopausal), BMI (continuous), benign breast lump (yes/no), history of breastfeeding (yes/no), family history of breast cancer in first degree relatives (yes/no) and duration of exposure to second-hand smoking (in years, continuous). | | | | | | | | | | | | |

| ***Supplementary table 4:*** Attributable fraction calculation | | | | | |
| --- | --- | --- | --- | --- | --- |
| **Factors** | **OR** | **Prevalence of chewing amongst cases** | **Proportion(P)** | **Attributable fraction (AF) Calculation [P x ((OR-1)/OR)]** | **AF** |
| Chewing tobacco | 1.19 | 16.76 | 0.1676 | 0.026759664 | 2.68 |

| ***Supplementary table 5:*** Population attributable fraction calculation | | | | | | |
| --- | --- | --- | --- | --- | --- | --- |
| **Sr. No.** | **State/union territory** | **Prevalence (%)*** | **Proportion (P)** | **OR for tobacco chewing from study** | **PAF [P x (OR-1)] / [P x (OR-1) + 1]** | **PAF%** |
| 1 | Chandigarh | 0.8 | 0.008 | 1.19 | 0.002 | 0.15 |
| 2 | Delhi | 3.2 | 0.032 | 1.19 | 0.006043257 | 0.60 |
| 3 | Haryana | 2.2 | 0.022 | 1.19 | 0.0041626 | 0.42 |
| 4 | Himachal Pradesh | 0.1 | 0.001 | 1.19 | 0.000189964 | 0.02 |
| 5 | Jammu& Kashmir | 1.5 | 0.015 | 1.19 | 0.002841901 | 0.28 |
| 6 | Ladakh | NA | NA | 1.19 | NA | NA |
| 7 | Punjab | 0.3 | 0.003 | 1.19 | 0.000569675 | 0.06 |
| 8 | Rajasthan | 5.8 | 0.058 | 1.19 | 0.010899883 | 1.09 |
| 9 | Uttarakhand | 3.4 | 0.034 | 1.19 | 0.006418536 | 0.64 |
| 10 | Chhattisgarh | 24.5 | 0.245 | 1.19 | 0.04447948 | 4.45 |
| 11 | Madhya Pradesh | 16.8 | 0.168 | 1.19 | 0.03093263 | 3.09 |
| 12 | Uttar Pradesh | 15.2 | 0.152 | 1.19 | 0.028069357 | 2.81 |
| 13 | Bihar | 3.6 | 0.036 | 1.19 | 0.006793532 | 0.68 |
| 14 | Jharkhand | 15.7 | 0.157 | 1.19 | 0.028965946 | 2.90 |
| 15 | Odisha | 33.6 | 0.336 | 1.19 | 0.060009024 | 6.00 |
| 16 | West Bengal | 17.2 | 0.172 | 1.19 | 0.031645815 | 3.16 |
| 17 | Arunachal Pradesh | 27.7 | 0.277 | 1.19 | 0.049998575 | 5.00 |
| 18 | Assam | 32.5 | 0.325 | 1.19 | 0.0581587 | 5.82 |
| 19 | Manipur | 45.2 | 0.452 | 1.19 | 0.079087929 | 7.91 |
| 20 | Meghalaya | 29.1 | 0.291 | 1.19 | 0.052393181 | 5.24 |
| 21 | Mizoram | 46 | 0.46 | 1.19 | 0.080375207 | 8.04 |
| 22 | Nagaland | 31.5 | 0.315 | 1.19 | 0.056470255 | 5.65 |
| 23 | Sikkim | 5.1 | 0.051 | 1.19 | 0.009597005 | 0.96 |
| 24 | Tripura | 56.5 | 0.565 | 1.19 | 0.096943153 | 9.69 |
| 25 | Dadra & Nagar Haveli | NA | NA | 1.19 | NA | NA |
| 26 | Daman & Diu | NA | NA | 1.19 | NA | NA |
| 27 | Goa | 3.6 | 0.036 | 1.19 | 0.006793532 | 0.68 |
| 28 | Gujarat | 10 | 0.1 | 1.19 | 0.018645731 | 1.86 |
| 29 | Maharashtra | 16.6 | 0.166 | 1.19 | 0.030575644 | 3.06 |
| 30 | Andaman & Nicobar Islands | NA | NA | 1.19 | NA | NA |
| 31 | Andhra Pradesh | 6.6 | 0.066 | 1.19 | 0.012384696 | 1.24 |
| 32 | Karnataka | 10.3 | 0.103 | 1.19 | 0.019194366 | 1.92 |
| 33 | Kerala | 3.6 | 0.036 | 1.19 | 0.006793532 | 0.68 |
| 34 | Lakshadweep | NA | NA | 1.19 | NA | NA |
| 35 | Puducherry | 4.9 | 0.049 | 1.19 | 0.009224123 | 0.92 |
| 36 | Tamil Nadu | 9.3 | 0.093 | 1.19 | 0.017363192 | 1.74 |
| 37 | Telangana | 9 | 0.09 | 1.19 | 0.016812506 | 1.68 |
| Overall prevalence in India |  | 12.8 | 0.128 | 1.19 | 0.02374258 | 2.37 |
|  |  |  |  |  |  |  |
| *Abbreviations used: NA is Not available, OR is Odds Ratio.  *Prevalence taken form GATS-2 (2016-17), for use of smokeless tobacco among women.* | | | | | | |

***
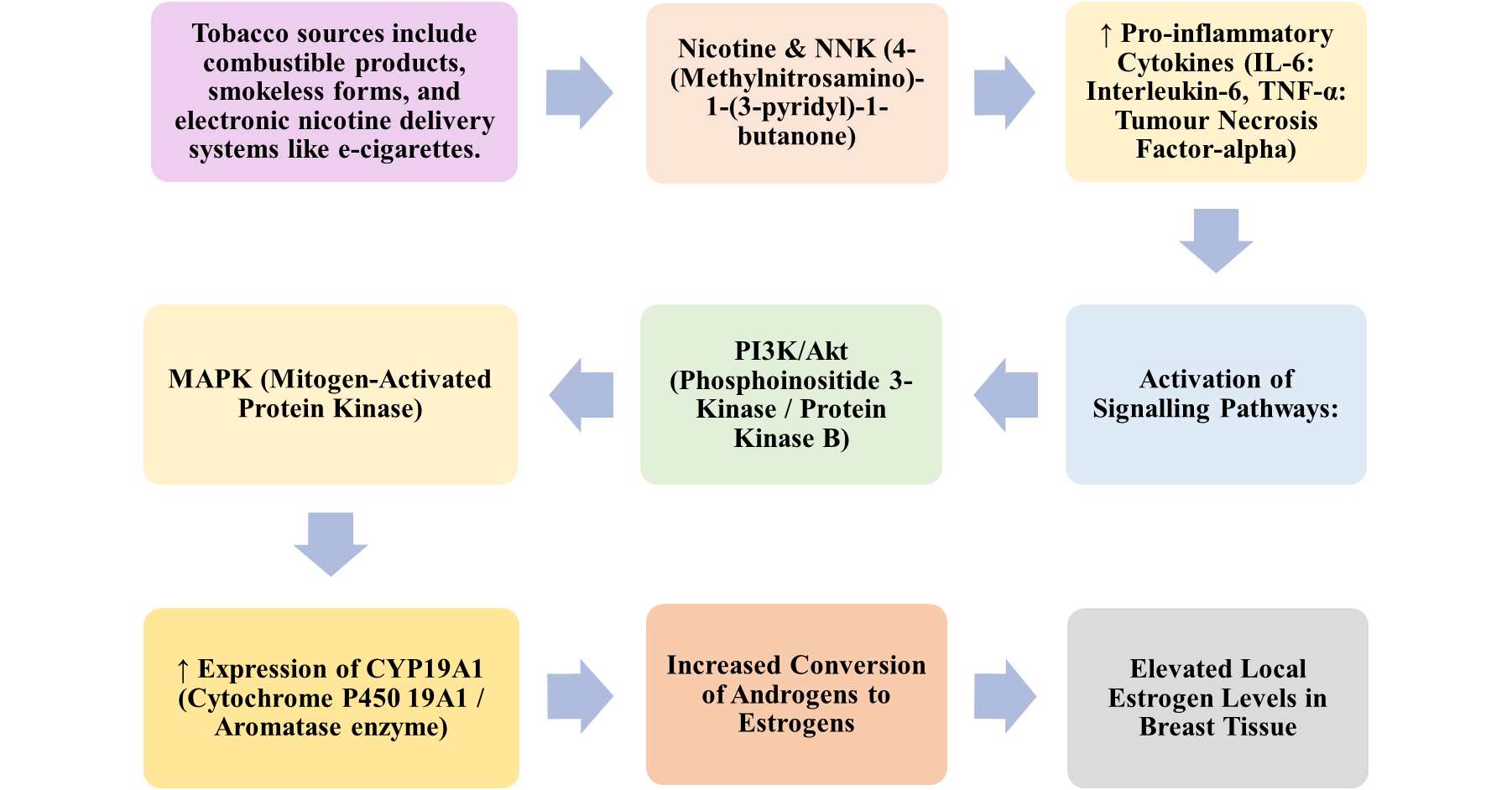
Supplementary figure 3*:** Flowchart showing how nicotine and NNK trigger inflammation and activate PI3K/Akt and MAPK pathways

***Supplementary figure 4****:* Graphical representation of sample size required to estimate different levels of odds ratio


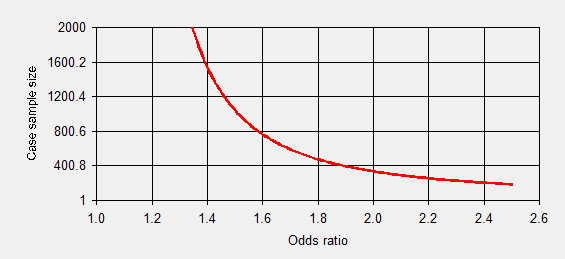


For a study with 1 control(s) per case and prior data from National Family Health Survey (NFHS-5) indicating that the prevalence of chewing tobacco among Indian females to be ~9%, in order to estimate true odds ratio for disease to be 1.65, we will need to study 670 case patients and 670 control patients.

**Note: Power has been considered 80% and the type I error probability has been considered 5%.*

**Description of supplementary materials:**

1. ***Supplementary figure 1*:** This contains inclusion and exclusion criteria for cases and controls.
2. ***Supplementary table 1:*** Shows the distribution of controls in the different cancer Disease Management Groups (DMGs).
3. ***Supplementary figure 2:*** This contains the image of the section (Chewing tobacco) of the questionnaire which was used to collect information regarding chewing tobacco for current study.
4. ***Supplementary table 2:*** Shows the summary characteristics of study participants based on their hormone receptor status.
5. ***Supplementary table 3:*** Shows the odds ratio for association chewing tobacco and the risk of breast cancer, which has been stratified for BMI.
6. ***Supplementary table 4:*** Shows the attributable fraction (AF) of breast cancer due to tobacco chewing in our study population, along with the description of formula used for calculation.
7. ***Supplementary table 5:*** Shows the population attributable fraction (PAF) of breast cancer due to tobacco chewing in various states of India.
8. ***Supplementary figure 3:*** This figure contains a flowchart showing how nicotine and NNK trigger inflammation and activate PI3K/Akt and MAPK pathways.
9. ***Supplementary figure 4:*** This figure contains graphical representation of sample size required to estimate different levels of odds ratio along with description of how it was calculated.
